# The Potential Contribution of Dysfunctional RNA-Binding Proteins to the Pathogenesis of Neurodegeneration in Multiple Sclerosis and Relevant Models

**DOI:** 10.3390/ijms21134571

**Published:** 2020-06-27

**Authors:** Cole D. Libner, Hannah E. Salapa, Michael C. Levin

**Affiliations:** 1 Department of Health Sciences, University of Saskatchewan, Saskatoon, SK S7N 5A2, Canada; cole.libner@usask.ca; 2 Office of Saskatchewan Multiple Sclerosis Clinical Research Chair, CMSNRC (Cameco MS Neuroscience. Research Center), University of Saskatchewan, Saskatoon, SK S7K 0M7, Canada; hes763@mail.usask.ca; 3 Department of Medicine, Neurology Division, University of Saskatchewan, Saskatoon, SK S7N 5A2, Canada

**Keywords:** neurodegeneration, multiple sclerosis, RNA-binding proteins, hnRNP A1

## Abstract

Neurodegeneration in multiple sclerosis (MS) is believed to underlie disease progression and permanent disability. Many mechanisms of neurodegeneration in MS have been proposed, such as mitochondrial dysfunction, oxidative stress, neuroinflammation, and RNA-binding protein dysfunction. The purpose of this review is to highlight mechanisms of neurodegeneration in MS and its models, with a focus on RNA-binding protein dysfunction. Studying RNA-binding protein dysfunction addresses a gap in our understanding of the pathogenesis of MS, which will allow for novel therapies to be generated to attenuate neurodegeneration before irreversible central nervous system damage occurs.

## 1. Neurodegeneration in Neurologic Disease of the Central Nervous System

Neurodegeneration is a frequently used term to describe many neurological diseases, but its precise definition varies. Taken literally, neurodegeneration is the breakdown of the nervous system, a very broad understanding of the term that is open to interpretation. It may be defined as the death or damage to cells of the nervous system which may include neurons, oligodendrocytes, and other glial cells. Alternatively, it can be defined as the death or damage exclusively to neurons and their axons, as a functional unit of the nervous system. In this review neurodegeneration is defined as the death and damage to central nervous system (CNS) neuronal cell bodies and their axons. In diseases with a neurodegenerative component, the death of neurons is not usually the direct cause of mortality, but rather facilitates the occurrence of secondary health problems [1]. Common neurologic diseases that contain a substantial neurodegenerative component include Alzheimer’s disease (AD), Parkinson’s disease (PD), amyotrophic lateral sclerosis (ALS), frontotemporal lobe dementia (FTLD), Huntington’s disease (HD), and multiple sclerosis (MS). This review provides the most current knowledge and gaps in our understanding of the mechanisms underlying neurodegenerative processes occurring in MS and its models.

## 2. Etiology and Clinical Features of MS

MS is a demyelinating, autoimmune, and neurodegenerative disease of the CNS. It affects more than 2.2 million people world-wide [2]. MS is a severe debilitating disease that results in a variety of physical and cognitive deficits. The etiology of MS is largely unknown; however, it is believed that an environmental factor combined with genetic predisposition triggers an autoimmune response to CNS antigens. Viral infections have long been considered a crucial environmental factor contributing to MS pathology [3]. Epstein–Barr virus, a virus mainly targeting B cells, is the best studied [4,5,6], but thus far an MS-specific viral infection has not been identified. Over 200 risk loci for MS have been identified using genome-wide association studies. The majority of the risk loci are associated with immune system components, including genes involved in cytokine pathways, signal transduction, and costimulatory molecules [7]. The human leukocyte antigen (HLA) genes, particularly class 2 HLA-DR2 (HLA-DRB1*1501), are the most strongly associated with MS disease risk [8,9].

The majority of MS patients initially present with a relapsing remitting disease course, where relapses of clinical symptoms are followed by a period of remission in which symptoms partially or completely recover. The clinical manifestations of relapses are considered to be a result of acute inflammation and demyelination [10]. Over time, a significant proportion of relapsing-remitting MS patients will develop a progressive form of the disease (secondary-progressive MS) involving continuous worsening of neurological symptoms. A small percentage of patients present initially with primary-progressive MS where disease continuously progresses with no relapsing phase. Ongoing neurodegeneration is characteristic of progressive disease and is believed to be responsible for the permanent, irreversible damage in MS patients [11,12,13]. Neurodegeneration underlies permanent disability in MS patients, but its initiation is difficult to define as it may occur prior to symptom onset. At early stages, redundancy in the CNS can compensate when small numbers of neurons or axons are damaged, which is undetectable symptomatically. The loss of a larger number of neurons or axons leads to the manifestation of clinical symptoms. The clinical significance of neurodegeneration is important because it has a stronger correlation with clinical disability than demyelination [14,15,16,17]. Gray matter damage, degeneration of neuronal cell bodies, and axonal transection in the brains of MS patients is known to occur early in disease and can be dependent or independent from inflammation or demyelination [12,13,18,19,20,21].

Most of the treatments for MS are immunotherapies that act to regulate an overactive immune system. These drugs have been proven to be effective in offering a better quality of life for the majority of patients, specifically those with relapsing-remitting MS [22,23]. A primary limitation of these immunotherapies is the downregulation of the immune system, which puts patients at risk of developing comorbidities such as infections and cancer. The reason why these therapies might not significantly attenuate progressive disease is because they fail to disrupt the neurodegenerative process [24]. Currently, there is a gap in our understanding of the neurodegenerative processes occurring in MS, creating an urgent need to identify early detectable markers of neurodegeneration along with the mechanisms underlying it so effective therapeutics can be developed to inhibit progression and irreversible damage in MS.

## 3. Mechanisms of Neurodegeneration in MS and Its Models

The cause of neurodegeneration in MS is predominately unknown, but is likely complex and multifactorial. Many mechanisms have been proposed and researched in depth, such as the increased energy requirements, sodium and calcium channel redistribution, and cellular stress that results from the demyelination of axons [25,26,27]. This review focuses on mechanisms of neurodegeneration resulting from mitochondrial dysfunction, oxidative stress, and neuroinflammation, and provides a summary of data suggesting that dysfunctional RNA-binding proteins may contribute to MS pathogenesis of neurodegeneration in MS.

### 3.1. Mitochondrial Dysfunction and Oxidative Stress

The brain utilizes roughly 20% of the total energy of the body, illustrating the importance and extremely high energy demands of the CNS [28,29]. In addition to producing energy, mitochondria also function to produce amino acids, maintain calcium homeostasis, and modulate reactive oxygen species (ROS) levels and thus, oxidative stress. Perturbation of mitochondrial processes, therefore, can lead to energy insufficiencies, disruptions in cellular transport, release of ROS and resultant oxidative stress, altered ion channel dynamics and subsequent axonal degeneration and necrosis in the CNS [30]. Postmortem analysis of neurons from progressive MS patients has demonstrated decreased activity of neuronal mitochondrial respiratory chain complexes I and III in the motor cortex indicative of severe mitochondrial dysfunction [31]. This suggests that ATP production may be decreased, which could negatively impact axonal and neuronal survival due to unmet energy demands. Another study using EAE further supports a relationship between mitochondrial dysfunction and neurodegeneration by demonstrating that axonal mitochondrial dysfunction is present prior to EAE disease onset and parallels neurological deficits [29]. In addition, mitochondrial dysfunction in neurons was associated with mitochondrial fragmentation, decreased mitochondrial mobility and decreased membrane potential suggestive of axonal transport deficiencies, a marker of neurodegeneration. Detoxification using scavengers that reduce ROS and oxidative stress was found to reverse mitochondrial pathology and rescue axons from degeneration in EAE indicating that mediating the consequences of mitochondria dysfunction might reverse neurodegeneration [32]. Further studies utilizing antioxidant therapy in EAE to combat the effects of mitochondrial dysfunction have yielded promising results, but data from human clinical trials has shown mixed results [33]. For example, antioxidant therapies, such as vitamin D3 supplementation, in patients with MS demonstrated beneficial results [34,35]. A double-blinded randomized pilot trial found that taking a high dose of lipoic acid, an endogenously-produced antioxidant, slowed the whole brain atrophy rate in secondary-progressive MS [36], suggesting that combating oxidative stress can prevent neurodegeneration. However, other antioxidant therapies, including eicosapentaenoic acid and docosahexaenoic acid, provided no benefit [37,38].

### 3.2. Neuroinflammation

In the CNS, neuroinflammation is initiated as a protective response, but its chronic presence results in loss of trophic support by glial cells and has detrimental effects on neurons. Innate immune system cells, including CNS-resident microglia and astrocytes, endothelial cells, and immune cells that infiltrate from the periphery [39] produce a number of chemokines, cytokines, and ROS, resulting in a toxic environment that contributes to the onset and progression in neurologic diseases, such as AD, PD, and MS [40]. Neuroinflammatory environments, including those within MS, have been shown to contribute to neurodegeneration, leading to further exacerbation of neuroinflammation [41] and resulting in an ongoing cycle of CNS damage.

Emerging themes in neuroinflammation suggest microglial activation is a heterogeneous process and can result in either a proinflammatory cytotoxic M1 phenotype that can contribute to neuronal damage or an immunosuppressive and neuroprotective M2 phenotype [42]. A greater number of proinflammatory M1 microglia are found in neurodegenerative conditions compared to the anti-inflammatory M2 microglia [42]. M1 microglia likely contribute to neuronal damage by releasing proinflammatory cytokines and molecules, proteolytic enzymes, and free radicals [43]. Macrophages and microglia also produce ROS, leading to increased oxidative stress, dysfunctional mitochondrial activity, including destabilization of the mitochondrial membrane [30,44], and subsequent neuronal and axonal damage.

The adaptive immune response, including both T and B lymphocytes, has been shown to play a role in the pathogenesis of MS. Several studies have shown that T cells contribute to neuronal and axonal damage in MS and its models [45,46,47,48,49,50,51,52]. For example, a study using in vivo live two-photon imaging of mice with EAE showed a direct interaction between myelin oligodendrocyte glycoprotein (MOG)-specific Th17 cells and neuronal cell bodies and axons in the spinal cord [53]. This interaction between immune and CNS cells was associated with extensive axonal damage [53]. Additionally, these interactions resulted in increased Ca^2+^ in neuronal cell bodies, which preceded axonal transection. Further studies have found a relationship between leptomeningeal inflammation in secondary-progressive MS cases and increased subpial cortical demyelination, cortical microglial activation, and neurodegeneration [54,55,56,57,58]. Neuronal loss was not confined to cortical lesions, but was also present in the normal-appearing gray matter adjacent to leptomeningeal inflammation. Areas containing leptomeningeal inflammation were found to contain many ectopic lymphoid structures, which contained plasmablasts, T cells, and dendritic cells, suggesting a role for the adaptive immune system in gray matter damage [53,57,59,60,61].

The presence of plasmablasts in these areas also implicates antibodies in MS pathogenesis. MS has been considered an antibody-mediated disorder of the CNS due to the presence of IgG oligoclonal bands detected in the cerebrospinal fluid (CSF) of greater than 90% of MS patients [60]. Antibodies to myelin antigens, such as myelin oligodendrocyte glycoprotein (MOG), myelin basic protein (MBP), and proteolipid protein (PLP), have been identified [61,62,63]. These antibodies have been shown to play a primary role in the demyelination of axons [62,64] through complement cascade activation [65,66]. Interestingly, antibodies to nonmyelin antigens such as neurofascin, neurofilament, and heterogeneous nuclear ribonucleoprotein A1 (hnRNP A1), a RNA-binding protein (RBP), seem to contribute to axonal and neuronal injury [67,68,69,70,71].

## 4. RNA-Binding Proteins

### 4.1. RNA-Binding Proteins and Stress Granules in Neurodegenerative Diseases

RBPs are highly conserved proteins whose expression is tightly regulated and necessary for cell survival. They play important roles in the regulation of gene expression as well as mRNA stability, splicing, and transport. Under homeostatic conditions, many RBPs localize to the nucleus where they continuously shuttle back and forth to the cytoplasm; a process mediated by nuclear export and nuclear localization signals within the RBP. RBPs contain functional regions that have the ability to bind RNA as well as other proteins. For example, hnRNP A1 includes two RNA binding domains located in the N-terminal half of the molecule, a glycine-rich C-terminal containing the RGG box, RGG domain and the M9 shuttling domain [69]. The glycine rich C-terminus, also known as a low complexity domain (LCD), mediates liquid–liquid phase separation (LLPS) [72]. LLPS is the conversion of a homogenous solution into a dense phase mediated by RNA–RNA, RNA–RBP and RBP–RBP multivalent interactions, and a dilute phase (the cytoplasm). Under homeostatic circumstances, LLPS allows for the concentration of certain RNAs and RBPs into membraneless structures such as stress granules (SGs), which generally contain a denser core and a more dynamic shell that interacts with the surrounding cytoplasm [73].

SGs have been tightly linked to neurodegeneration in human neurologic diseases as well as their models [70,74,75]. In response to stress, cells initiate the integrated stress response, an initiative to protect the cell [76]. The first step of the integrated stress response is the phosphorylation of eukaryotic translation initiation factor 2α (eIF2α), slowing translation of mRNAs and promoting the formation of higher-order structures including SGs [77]. Cytoplasmic SGs are dynamic, membraneless granules that harbor translationally repressed mRNAs and proteins, including RBPs, as a mechanism of protection. SG formation alters patterns of local RNA translation facilitating the stress response [78]. Under physiological conditions, once the acute stressor is removed, SGs disassemble and translation resumes. In contrast, under pathologic conditions, SGs may persist and have been shown to lead to decreased cell survival and the induction of proapoptotic pathways [79,80,81,82]. Augmented SG formation has also been shown to decrease cell viability [80]. Abnormally stable SGs may serve as a platform for formation of larger protein aggregates, and further interactions between RNA and RBPs within SGs may change LLPS dynamics, leading to a lack of disassembly [83]. The prolonged sequestration of important RBPs and mRNAs needed for cell survival combined with translational impairment might also be a mechanism of cell death [84]. A recent study in neuronal cells demonstrated that in response to stress there was a significant correlation between the proteins being actively translated and those transcripts depleted from SGs, including proteins associated with neurodegenerative diseases [85]. Further, it was found that mutant RBPs within SGs can further alter neuronal gene expression [85]. Significant advances in identifying the components and structure of SGs was reviewed by Youn et al. [86]. This review is supported by a new user-friendly database that searches the current literature evidence for genes and proteins that associate with SGs [86]. Lastly, they provide an interpretation of how components of SGs interact to form these membraneless structures [86]. How components and structures of SGs in MS compare to those in other neurologic diseases remains a topic for future study.

Considering the functional importance of RBPs in neurons as well as their ability to form higher order structures through LLPS, recent data implicate RBP dysfunction as a pathogenic hallmark of ALS, FTLD, and most recently MS [87,88,89,90]. Dysfunctional RBPs, including hnRNP A1, Tar-DNA binding protein-43 (TDP-43), and Fused in sarcoma (FUS), have been shown to result in a triad of molecular changes including RBP mislocalization from its normal (homeostatic) nuclear location to the cytoplasm, SG formation, and altered RNA metabolism [91,92], all of which contribute to neurodegeneration.

The implications of dysfunctional RBPs for neuronal health are multifactorial. RBP mislocalization could result in both gain of toxicity in the cytoplasm and loss of functionality in the nucleus. Decreased RNA availability in the cytoplasm can cause RBPs to become more aggregate-prone due to their LCDs, resulting in the potential to create cytoplasmic inclusions [93,94]. Under pathogenic conditions, and where decreasing concentrations of RNA within the cytoplasm combined with mislocalized RBPs, LLPS may be driven towards a less dynamic and more aggregate-prone phase, which can lead to cell toxicity [93,95]. ALS mouse models exhibiting cytoplasmic FUS gain of toxicity mechanisms show progressive motor neuron degeneration [96]. In addition, increased cytoplasmic RBP mislocalization has been tied to more severe disease phenotypes in mouse models as well as humans [75,97,98]. Furthermore, cytoplasmic TDP-43 leads to the abnormal binding and splicing of cytosolic RNA targets [99,100]. Counter to cytoplasmic consequences, decreased or depleted RBP availability in the nucleus can lead to improper RNA processing, including disrupted splicing and impaired transcription control [101]. In fact, TDP-43 nuclear depletion has been shown to be sufficient to induce neuronal cell death [102]. A recent study demonstrated that TDP-43 overexpression or knockdown results in similar changes in mRNA abundances and morphological defects, including reduced dendritic branching of neurons [103]. Targets of TDP-43 have been identified to be enriched for pathways involving the transcription factor cAMP response element-binding protein (CREB) [104], which is important for neurogenesis, neuronal survival, plasticity, and differentiation [105,106]. CREB activation is inhibited by TDP-43 dysfunction, while restoring CREB rescued defects in dendritic branching [104]. Dysfunctional RBPs and disruption of SG dynamics can lead to pathogenic SGs that persist and become cytotoxic [74]. Several studies have demonstrated that inhibiting SG formation suppresses nucleocytoplasmic mislocalization of RBPs and neurodegeneration in ALS and FTLD models [107].

Several factors have been found to disrupt RBP function, including mutations within RBPs, osmotic stress, oxidative stress, heat stress, and proinflammatory cytokines [75,108,109]. For example, in vitro experiments demonstrated that somatic mutations within hnRNP A1 identified from MS patients contribute to RBP dysfunction [110]. These experiments showed that transfection of plasmids containing hnRNP A1 mutants in neuron-like cells resulted in hnRNP A1 mislocalization, colocalization into cytoplasmic SGs, and apoptosis, compared to cells transfected with plasmids containing wild type hnRNP A1. Considering inherited mutations within RBPs and subsequent RBP dysfunction were shown to underlie ALS and FTLD pathology [74,88,96,111,112,113], these experiments suggest similar mechanisms may contribute to the pathogenesis of MS and like inherited mutations, somatic mutations may be amenable to Clustered Regularly Interspaced Short Palindromic Repeats-Cas9 (CRISPR-Cas9) therapy. Other experiments have shown that oxidative stress and subsequent DNA damage associated with Poly ADP-ribose polymerase (PARP) activation in the brain lead to the accumulation of dysfunctional RBPs in neurons [114,115]. Extensive activation of PARP is related to cell death while inhibition has been found to attenuate inflammation and improve neuronal survival [116]. PARP can also induce Poly ADP-ribosylation (PARylation), a major regulator of SG assembly and disassembly [117]. For example, PARylation of hnRNP A1 contributes to its nucleocytoplasmic mislocalization and association within SGs, while inhibition of PARP was found to mitigate hnRNP A1 mediated neurotoxicity [117]. Additionally, oxidative stress induced by 3-nitropropionic acid toxin, a mitochondrial respiratory complex II inhibitor, has been shown to lead to hnRNP A1 mislocalization from nucleus to cytoplasm in rodents [118]. Cytoplasmic hnRNP A1 further induced mitochondrial dysfunction, leading to a brain-focused pattern of cell death in animals [118,119].

### 4.2. RNA-Binding Proteins in MS

The previously mentioned data demonstrate that dysfunctional RBP biology, including RBP mislocalization, SG formation, and subsequent changes in RNA metabolism, can contribute to neurodegeneration in numerous neurological conditions. More recent data indicates that dysfunctional RBP biology may play a role in MS pathogenesis and contribute to neurodegeneration in a manner similar to other neurologic diseases.

#### 4.2.1. Evidence in Glia and Immune Cells

Because MS involves the immune system in addition to the CNS, it is prudent to consider the effects that changes in RBPs in immune cells and glia, such as astrocytes and microglia, might play in disease. For example, differential expression of RBPs, including HuR (Human antigen R) and TDP-43, has been identified in PBMCs and T-cells, isolated from MS patients [120,121], suggesting that these RBPs are dysregulated in immune cells although how this influences disease is unclear. Further studies have demonstrated that an inflammatory environment, such as in MS, can lead to the mislocalization of RBPs in astrocytes and microglia and can result in the recruitment of additional inflammatory molecules, which can be deleterious to neurons [122,123]. These data highlight the role of RBPs in glial cells and how disruption of normal RBP functions in these cells may be harmful. Additionally, the proinflammatory factors secreted by immune cells in a potent inflammatory environment may also drive RBP dysfunction. For example, TNFα and IFNγ, potent cytokines that have been shown to be increased in MS patient cerebrospinal fluid samples [124,125], have been shown to induce RBP dysfunction in neuronal cells [108,122]. Exposure of neuronal cells IFNγ was found to induce RBP dysfunction, including hnRNP A1 mislocalization, altered SG dynamics, and changes in RNA metabolism, suggesting that proinflammatory factors can negatively influence RBPs [108].

The role of RBPs in oligodendrocytes has also been investigated. Interestingly, TDP-43 mislocalization and aggregate formation were found in both oligodendrocytes and anterior horn cells in mice infected with Theiler’s murine encephalomyelitis virus, another animal model of MS [126]. These findings are particular relevant as other studies have shown that deletion of TDP-43 in oligodendrocytes in mice results in neurological deficits as they age [127] suggesting that proper functioning of TDP-43 is crucial to oligodendrocyte survival and function. Myelin-associated mRNAs have also been shown to be sequestered in SGs in oligodendrocytes in vitro under times of stress [128]. Prolonged sequestration of pertinent myelin-associated mRNAs in SGs could have detrimental effects on myelination processes, including maintenance. Together these data suggest that dysfunctional RBP biology, which is observed in glial cells in models of MS, can negatively impact oligodendrocyte health and function, leading to deleterious effects.

#### 4.2.2. Evidence in Neurons

Although changes in RBPs have been observed in glia and immune cells and have been associated with the secretion of proinflammatory factors in MS and important in vitro models, this does not directly implicate dysfunctional RBPs in neurodegeneration. However, there is substantial evidence demonstrating RBP dysfunction in neurons in MS and its models suggesting this may be an underlying mechanism of neurodegeneration. For example, hnRNP A1 mislocalization and SG formation, two of the pathological hallmarks of dysfunctional RBPs, were initially shown in neurons from a single MS case [108]. Recently, these findings were expanded to include 12 additional MS cases and six control cases, and demonstrated increased nucleocytoplasmic mislocalization of hnRNP A1 and TDP-43 in neurons from MS patients compared to controls [129]. Increased nucleocytoplasmic localization of RBPs has been shown to be toxic to neurons [91,101]. In a separate study, altered RBP biology was identified in oligodendrocytes and neurons of MS cases. These data found altered expression of the RBPs TDP-43 and polypyrimidine tract-binding protein 1 and 2 (PTB1 / PTB2) in cortical demyelinated lesions [130]. Considering the importance of TDP-43 and PTB1/2 in oligodendrocyte viability and neuronal differentiation, respectively, researchers hypothesized RBP dysfunction might contribute to cortical lesion damage and neurodegeneration in MS [130].

**Figure 1:**
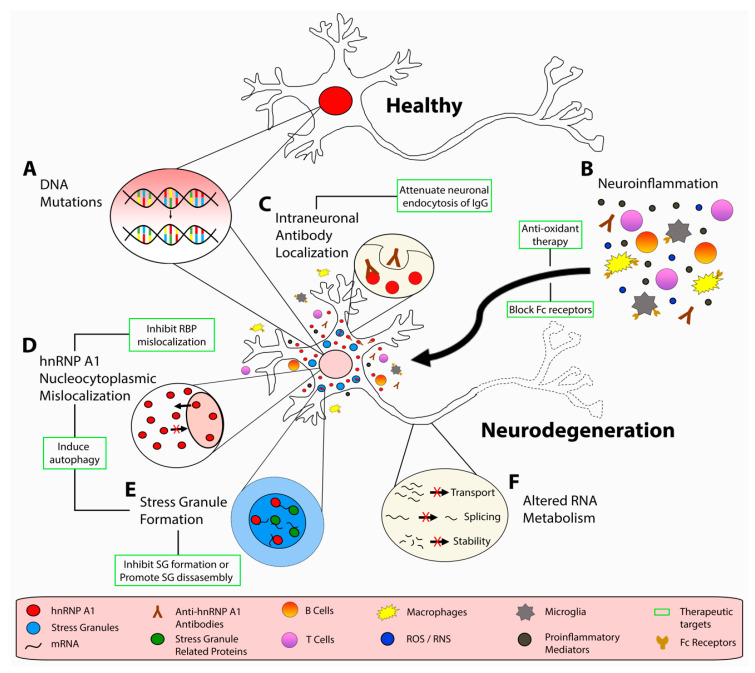
Potential pathogenic mechanisms of neurodegeneration in multiple sclerosis (MS). Under, homeostatic conditions, hnRNP A1 (red) is localized to the nucleus of neuronal cell bodies. In response to (**A**) genetic mutations, (**B**) neuroinflammation and corresponding proinflammatory mediators, oxidative stressors, and (**B**,**C**) autoantibodies, neuronal cell bodies display dysfunctional hnRNP A1 biology, including (**D**) hnRNP A1 nucleocytoplasmic mislocalization, (**E**) stress granule formation, and (**F**) altered RNA metabolism. A number of therapeutic strategies have been identified, which may prevent dysfunctional RBP-mediated neurodegeneration. These strategies include using (**B**) antioxidant and/or Fc blocking therapies, (**C**) altering neuronal endocytosis of IgG, (**D**) inhibition of RBP mislocalization, (**E**) inhibition of SG assembly or inducing SG disassembly, (**D**,**E**) and inducing autophagy.

Additional experiments from animal models of MS have provided evidence of dysfunctional RBPs contributing to neurodegeneration. Although an imperfect animal model of MS, experimental autoimmune encephalomyelitis (EAE) recapitulates certain aspects of MS pathology including demyelination, neuronal loss, and a robust CNS immune response. Potential drawbacks of the EAE model are variability between mouse strains and immunizing agent along with favoring a CD4^+^ T cell response, whereas in MS, CD8^+^ T cells and B cells are known to play an important role. Nevertheless, EAE mice showed increased hnRNP A1 and TDP-43 mislocalization and SG formation in neurons of the spinal cord, the former of which correlated with neurodegeneration [70,75]. Additionally, hnRNP A1 mislocalization was found to correlate with disease severity as well as with IFNγ-producing CD3+ T cell infiltrates in the spinal cord of mice with EAE [75]. Furthermore, there was a significant negative correlation between the number of neurons in the spinal cord and hnRNP A1 mislocalization, indicating that there are fewer neurons in areas with increased hnRNP A1 mislocalization [75]. These experiments suggest a relationship between neurodegeneration, inflammation, and RBP dysfunction (Figure 1).

Other experiments have employed techniques to correct RBP dysfunction in EAE to determine the role it may play. KPT-350, a nuclear export inhibitor, that has been shown to correct RBP mislocalization phenotypes in other disease models, decreased disease severity, neurodegeneration, and demyelination when administered to EAE animals; however, its effect on RBP mislocalization in EAE was not examined [131]. Additionally, blocking the activity of the RBP HuR in EAE animals improved motor function and decreased demyelination, suggesting that RBPs contribute to clinical disease severity in EAE [132]. Furthermore, rapamycin, an autophagy inducer, that has been shown to correct TDP-43 mislocalization phenotypes and restore nuclear localization, reduced EAE severity, neuronal damage, and demyelination [133]. Finally, in EAE, poly ADP-ribose (PAR), a factor required for SG assembly [105,106], was found to be elevated in neurons, astrocytes, oligodendrocytes, and microglial in and around demyelinated plaques, suggesting increased SG formation, a feature heavily associated with dysfunctional RBPs [116]. Although these studies in MS and EAE do not explicitly demonstrate a mechanism of neurodegeneration through dysfunctional RBPs, they strongly suggest that RBP dysfunction is a feature of MS and its models, further implicating dysfunctional RBPs in neurodegeneration in a manner similar to other neurologic diseases.

#### 4.2.3. Autoimmunity to hnRNP A1 in MS

In addition to cytokine-induced mechanisms of RBP dysfunction, our lab has implicated an antibody response to hnRNP A1 as a mechanism of neurodegeneration in MS [69,70,87,134,135,136,137]. MS patients develop IgG specific for hnRNP A1, specifically to the M9 region of hnRNP A1, that is responsible for its transport into and out of the nucleus [69]. Previously published data from our lab demonstrated that peripheral injections of anti-hnRNP A1 antibodies which overlap with the immunodominant epitope of MS IgG, into mice with EAE resulted in worsening of disease, increased neurodegeneration, and a change in phenotype from flaccid to spastic hind limbs [71]. Spasticity of limbs is a common symptom of MS [138,139]. Mechanistically, our data suggest that antibodies to hnRNP A1 contribute to neurodegeneration both intra- and extra-neuronally. Immunohistochemical localization of anti-hnRNP A1 antibodies injected into mice with EAE showed antibody deposition within and surrounding spinal cord neurons. The antibodies surrounding neurons colocalized with Fc receptors on macrophages and induced nitric oxide synthase, responsible for increased nitric oxide production, an oxidative stressor. While all IgG are capable of binding Fc receptors, we have shown that compared to anti-hnRNP A1 antibodies, isotype control IgG did not localize to the spinal cord and did not colocalize with Fc receptors on macrophages [70,134]. To explain this, we demonstrated that in response to EAE, hnRNP A1 not only mislocalizes from the neuronal nucleus to the cytoplasm, but can also be found extra-neuronally, thus providing an antigenic target for anti-hnRNP A1 antibodies [70]. In contrast to anti-hnRNP A1 antibodies that target hnRNP A1, we believe isotype control IgG are not binding an antigenic target. Antibody deposition correlated with loss of spinal cord neuronal cell bodies, a marker of neurodegeneration. Although how anti-hnRNP A1 antibodies enter neurons and cause neuronal cell death is not yet clear, in vitro studies indicate clathrin-mediated endocytosis as a potential mechanism. For example, using neuron-like cell lines demonstrated that anti-hnRNP A1 antibodies entered neurons via clathrin-mediated endocytosis and resulted in a reduction of cellular ATP and increased expression of the apoptotic markers Caspase 3/7 [140,141]. Interestingly, antibodies to Tau protein have also been shown enter neurons via clathrin-mediated endocytosis [142]. These studies demonstrate the importance of autoimmunity in MS and its models, while also providing potential points of therapeutic intervention by blocking Fc receptors and/or altering neuronal endocytosis of pathogenic IgG (Figure 1).

Additionally, we found that peripheral injection of anti-hnRNP A1 antibodies also exacerbated hnRNP A1 dysfunction [70,71]. Anti-hnRNP A1 antibodies were localized to areas of the spinal cord with increased neuronal hnRNP A1 nucleocytoplasmic mislocalization, SG formation, and neuronal loss compared to controls. This study demonstrated that, in addition to the neuroinflammatory response in EAE, antibodies to hnRNP A1, an intraneuronal target, augments RBP dysfunction and neurodegeneration. We observed that neuroinflammation in EAE can cause extraneuronal localization of hnRNP A1 [70], thus exposing hnRNP A1 to the immune system as a neoantigen in our animal model and further providing evidence for a secondary autoimmune response. This may partially explain why MS patients, but not healthy controls, create autoantibodies to hnRNP A1.

## 5. Conclusions

Neurodegeneration in MS is likely due to a combination of mechanisms. Research suggests that dysfunctional RBPs are a pathologic hallmark of MS and its models and may contribute to neurodegeneration in mechanisms similar to other neurologic diseases. RBP dysfunction has been shown to be induced in response to a variety of stimuli, and in a number of cell types that lead to numerous downstream responses in neurons. In addition to RBP dysfunction, our work and the work of others show that neurodegeneration occurs as a result of neuroinflammation, autoimmunity, mitochondrial dysfunction and oxidative stress [70,75,88,108,110,111,112,113,122,143]. The contribution of different mechanisms of neurodegeneration might be explained by the heterogeneity of MS. Therefore, specific therapies will need to be tailored based on the predominant mechanism of neurodegeneration in an individual with MS. Further research involving dysfunctional RBPs is necessary to better understand the cellular pathways effected, so that precise therapeutic interventions can be created to prevent, attenuate, or reverse RBP dysfunction and in turn, alter the natural history of disease progression in MS.

## Abbreviations

AD	Alzheimer’s disease
ALS	Amyotrophic lateral sclerosis
cAMP	Cyclic adenosine monophosphate
CNS	Central nervous system
CREB	cAMP response element-binding protein
CSF	Cerebrospinal fluid
EAE	Experimental autoimmune encephalomyelitis
FTLD	Frontotemporal lobe dementia
FUS	Fused in Sarcoma
HD	Huntington’s disease
hnRNP A1	Heterogeneous nuclear ribonucleoprotein A1
HuR	Human antigen R
IFN	Interferon
LCD	Low complexity domain
LLPS	Liquid–liquid phase separation
MBP	Myelin basic protein
MOG	Myelin oligodendrocyte glycoprotein
MS	Multiple sclerosis
PAR	Poly ADP-ribose
PARP	Poly ADP-ribose polymerase
PBMCs	Peripheral blood mononuclear cells
PD	Parkinson’s disease
PLP	Proteolipid protein
PTBP	polypyrimidine tract-binding protein
RNS	Reactive nitrogen species
ROS	Reactive oxygen species
SG	Stress Granule
TDP-43	TAR-DNA binding protein-43
TNF	Tumor necrosis factor
